# Long-term in vivo expression of trastuzumab following intramuscular electrotransfer of the encoding DNA in mice

**DOI:** 10.1186/2051-1426-3-S1-O5

**Published:** 2015-08-14

**Authors:** Kevin Hollevoet, Nick Geukens, Greetje Vande Velde, Paul Declerck

**Affiliations:** 1 Laboratory for Therapeutic and Diagnostic Antibodies, KU Leuven - Leuven University, Leuven, Belgium; 2 PharmAbs, the KU Leuven Antibody Center - Leuven University, Leuven, Belgium; 3 Molecular Small Animal Imaging Center, KU Leuven - Leuven University, Leuven, Belgium

## 

In vivo antibody expression is at crossroads between monoclonal antibody (mAb) and gene therapy. Following a single intramuscular injection of the DNA that encodes a therapeutic mAb, the muscle is turned into a ‘bioreactor’, resulting in prolonged mAb secretion in circulation [1-3]. This innovative approach addresses several challenges associated with conventional mAb proteins. In R&D, in vivo mAb expression allows production and evaluation of leads directly into animal models. In the clinic, it can improve treatment efficacy and patient comfort by avoiding repeated high-dose injections, and provide a cost-effective answer to the increasing need for mAb combination therapies - in the field of cancer immunotherapy and beyond. This study outlines the development and delivery of a trastuzumab-encoding plasmid for in vivo mAb expression. To improve intramuscular DNA delivery in mice, we first established an optimal electrotransfer protocol using novel reporter plasmids. Following the optimized intramuscular electrotransfer of the trastuzumab-encoding plasmid, trastuzumab was detected with a commercial ELISA at therapeutically relevant concentrations (1-15 µg/ml) in the sera of athymic nude mice (n=16) for the full duration of the ongoing follow-up (>3 months). A cell viability assay demonstrated similar activity of the expressed versus commercial trastuzumab in the BT-4747 breast cancer cell line. In conclusion, we achieved proof of concept for the long-term in vivo expression of biologically active trastuzumab in mice. Ongoing work focuses on optimizing in vivo mAb expression for clinical application and evaluation for combination therapy.
